# A nomogram for predicting individual risk of acute kidney injury after endovascular therapy in large vessel occlusion stroke

**DOI:** 10.3389/fmed.2025.1608293

**Published:** 2025-10-08

**Authors:** Jingling Zhu, Xiaohua He, Wenfei Liang, Huishan Zhu, Jiasheng Zhao, Yu Ding, Xiuling Yang, Zhan Zhao, Jingyi Chen, Weimin Ning, Qiuxing He

**Affiliations:** ^1^Department of Neurology, Dongguan Hospital of Guangzhou University of Chinese Medicine, Dongguan, China; ^2^Dongguan Key Laboratory of Intractable Brain Diseases in Dongguan, Dongguan Hospital of Guangzhou University of Chinese Medicine, Dongguan, China; ^3^State Key Laboratory of Dampness Syndrome of Chinese Medicine, Dongguan Hospital of Guangzhou University of Chinese Medicine, Dongguan, China

**Keywords:** nomogram, clinical diagnosis prediction mode, acute kidney injury, acute ischemic stroke with large vessel occlusion, endovascular therapy

## Abstract

**Objective:**

This study was conducted to develop and validate a nomogram model for the early prediction of acute kidney injury (AKI) in patients with acute ischemic stroke with large vessel occlusion (AIS-LVO) following endovascular therapy (EVT).

**Methods:**

This retrospective study enrolled 450 patients with AIS-LVO admitted to the Dongguan Hospital of Guangzhou University of Chinese Medicine for EVT between July 2018 and September 2024. After applying exclusion criteria, 346 patients meeting the research criteria were included. These patients were randomly divided into a training cohort (*N* = 243) and a validation cohort (*N* = 103) at a 7:3 ratio for model development and validation. Least absolute shrinkage and selection operator (LASSO) regression and multinomial logistic regression analysis were employed for feature selection and identification of key predictors for the nomogram. The performance and clinical utility of the nomogram were assessed using the receiver operating characteristic (ROC) curve, calibration curve, clinical impact curve (CIC), and decision curve analysis (DCA) curve.

**Results:**

Hypertension, smoking, admission blood glucose, proteinuria, serum creatinine, and duration of mechanical ventilation were identified as independent risk factors for AKI in patients with AIS-LVO after EVT. The nomogram demonstrated excellent predictive performance, with an area under curve (AUC) of 0.890 [95% CI (0.846–0.935)]. These results indicate that the model offers a favorable net clinical benefit.

**Conclusion:**

The nomogram developed in this study demonstrates significant clinical utility in identifying patients with AIS-LVO at high risk of developing AKI after EVT.

## 1 Introduction

Endovascular therapy (EVT) has become the standard treatment for acute ischemic stroke with large vessel occlusion (AIS-LVO), significantly improving rates of vascular recanalization and neurological recovery (1–3). However, EVT is not without complications, and one often overlooked issue is renal organ damage following AIS-LVO surgery (4). The incidence of such complications ranges from 3.3 to 42.8%, depending on the population and clinical context (5–7). These events not only extend the intensive care unit (ICU) stays, imposing a substantial economic burden, but also prolong the duration of mechanical ventilation, and worsen patient prognosis and long-term outcomes (8, 9). Acute kidney injury (AKI) represents the earliest stage of renal dysfunction in this setting, characterized by a rapid decline in renal function (10, 11). The diagnosis of AKI is based on the Kidney Disease: Improving Global Outcomes (KDIGO) criteria, which define AKI by a progressive rise in serum creatinine (Scr) levels and/or a reduction in urine output over a short period (12). However, Scr levels only increase when the glomerular filtration rate (GFR) decreases by at least 70%, resulting in a delayed diagnosis of AKI—often 48–72 h after the onset of renal injury (13, 14). This delay in diagnosis frequently leads to missed opportunities for early intervention, increasing the risk of AKI progressing to chronic kidney disease (CKD) (15–17).

Once AKI occurs, few effective treatments are available to reverse renal dysfunction. Nonetheless, emerging evidence suggests that early restoration of renal function can reduce the likelihood of adverse renal events, underscoring the importance of timely interventions to improve long-term outcomes in AKI patients (18, 19). While prior studies have extensively investigated cardiorenal syndrome (CRS), stroke-induced renal dysfunction remains understudied. To date, no reliable predictive model exists to identify factors contributing to AKI in AIS patients undergoing EVT (20). Therefore, this study aims to address these gaps by investigating the incidence of AKI following EVT, identifying risk factors associated with its development, and evaluating the impact of AKI on the prognosis of AIS-LVO patients treated with EVT. Furthermore, we seek to develop a nomogram-based prediction model to facilitate early AKI diagnosis, providing critical insights for preventive and targeted therapeutic strategies.

## 2 Methods

### 2.1 Study design and participants

This study was a single-center retrospective study that enrolled 450 patients admitted to the Dongguan Hospital of Guangzhou University of Chinese Medicine for EVT between July 2018 and September 2024. After applying exclusion criteria, 346 patients who met the research criteria were included. A follow-up was conducted by phone or outpatient visit 90 days after the EVT. To obtain the prognosis, professional medical staff followed up with patients through a combination of telephone calls and outpatient visits, recording their recovery conditions and providing data for subsequent analysis of treatment effects.

The inclusion criteria were as follows: (1) All patients were diagnosed with ischemic stroke by CT or MRI scans, and CTA/MRA/DSA indicated AIS-LVO; (2) an age range of 18–90 years. The exclusion criteria were as follows: (1) Patients who were undergoing renal replacement therapy or had a history of renal transplantation; (2) Pregnant or lactating women; (3) Patients with incomplete medical records; (4) Patients who refused follow-up or were unreachable. After applying the exclusion criteria, 140 patients were excluded. Ultimately, 346 AIS-LVO patients were included. Subsequently, these patients were randomly divided into a training cohort (*N* = 243) and a validation cohort (*N* = 103) at a 7:3 ratio for model development and validation. Based on the Scr levels measured during the 7-day period after the EVT, the patients were classified into AKI and non-AKI group to thoroughly analyze the relevant data and probe into the factors influencing AKI following EVT. This study was conducted in accordance with the principles of the Declaration of Helsinki. The research was approved by the ethics committee of Dongguan Hospital of Guangzhou University of Chinese Medicine (PJ [2025] NO.5). Given that this retrospective observational study neither encroached on patient privacy nor presented any risk to patient safety, the Ethics Committee waived the requirement for informed consent. In order to safeguard patient confidentiality, the data derived from this study will not be released to the public.

### 2.2 Primary and secondary clinical outcomes

The primary outcome was the occurrence of AKI in AIS-LVO patients after EVT. AKI was defined according to the 2012 KDIGO Clinical Practice Guidelines (12).

Baseline Scr was the value measured within 24 h of admission and before EVT; it was used to calculate post-EVT changes. Because post-EVT diuretics required for volume control and intracranial-pressure management invalidate urine-output criteria, AKI was diagnosed exclusively by two Scr-based rules: (1) an absolute increase ≥26.7 μmol/L from baseline within 48 h, or (2) a ≥1.5-fold rise from baseline within 7 days after EVT. We implemented an Scr-monitoring protocol of intensive measurements during the first 48 h followed by routine sampling up to day 7. Early AKI was captured by 48-h re-measurements, and continued surveillance through day 7 identified delayed cases. AKI onset was defined as the first time that either criterion was met within the 7-day window, thereby accurately identifying EVT-associated AKI events.

The secondary outcome was the 90-day Modified Rankin Scale (mRS). We documented mRS scores and mortality rates. Patients were classified as having good (mRS 0–2) or poor (mRS 3–6, with 6 indicating death) functional outcome. All data were evaluated by three trained neurologists who had demonstrated good inter-rater agreement. Two physicians independently and blindly rated each patient; if their scores differed, the third rater adjudicated to obtain the final result.

Missing data (<10%) were handled by multiple imputation in SPSS. Sensitivity analyses confirmed that neither missing data nor the imputation method altered the core conclusions, and the model showed good robustness (Supplementary material 1).

### 2.3 Clinical data acquisition

In this research, we initially conducted a thorough and systematic search of the literature regarding the risk factors associated with AKI in patients following EVT. Drawing on the risk factors retrieved from the literature search and the accessible clinical and laboratory data of patients in the electronic medical record system, we pinpointed 30 potential variables. Meanwhile, we performed multicollinearity testing on these 30 variables using Stata software. The results demonstrated that the Variance Inflation Factor (VIF) of all variables was less than 10 (Supplementary material 2), indicating no significant multicollinearity among the variables. Thus, these variables can be stably incorporated into the multivariate model for subsequent analysis. During the data collection stage, we collected detailed demographic data of the patients from the electronic medical records, such as gender and age. Meanwhile, basic past medical history information was also collected, covering hypertension, diabetes, atrial fibrillation, heart failure, prior stroke, smoking, and alcohol drinking. In addition, professional neurologists rigorously evaluated the NIHSS score, mRS score, and GCS score when the patients were admitted to the hospital, and the results were fully recorded in the medical record system (21, 22). Drawing on previous research findings, we focused on data regarding postoperative complications, specifically intracranial hemorrhage, gastrointestinal hemorrhage, and brain herniation (23). In terms of clinical indicators, data such as proteinuria, Scr, cystatin C, uric acid, albumin, blood pressure at admission (systolic/diastolic), admission blood glucose, duration of mechanical ventilation, EVT operation time and ICU length of stay were collected (6). Because WBC, NLR, SII, PLR, SIRI and CRP reflect systemic inflammation and have been linked to post-stroke AKI, these indices were also included as candidate predictors.

### 2.4 Statistical analysis

All statistical analyses were completed using R Studio Statistics software (version 4.4.1), SPSS software (version 27.0), and Stata software (version 18.0). A two-tailed *p* < 0.05 was considered statistically significant. In the training cohort, all AKI-related feature variables were screened using the least absolute shrinkage and selection operator (LASSO). Subsequently, based on the screened feature variables, a clinical prediction model was established using multinomial logistic regression (stepwise backward regression). We utilized the selected predictors to build a nomogram to evaluate AKI risk. Eventually, the constructed model underwent validation by constructing the receiver operating characteristic (ROC) curve, calibration curve, decision curve analysis (DCA), and clinical impact curve (CIC). This was done to gage the predictive effectiveness and clinical application value of the model.

## 3 Results

### 3.1 Study flow diagram

**Figure 1:**
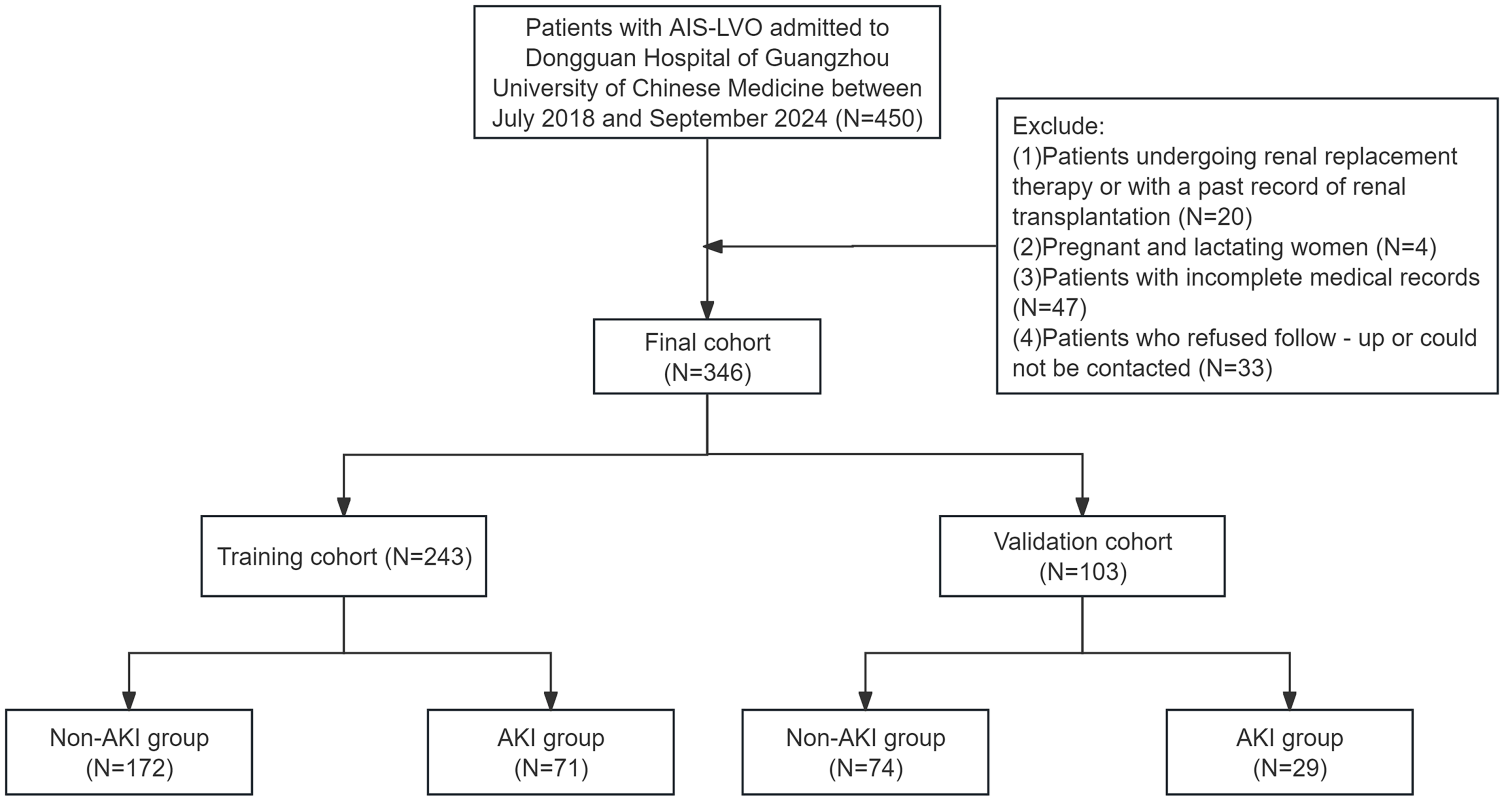
Flow chart of the data selection process.

The data selection flow chart is shown in Figure 1. Among 450 patients, 104 failed to meet the inclusion criteria. Eventually, a total of 346 patients participated in this study.

### 3.2 Patient characteristics

**Figure 2:**
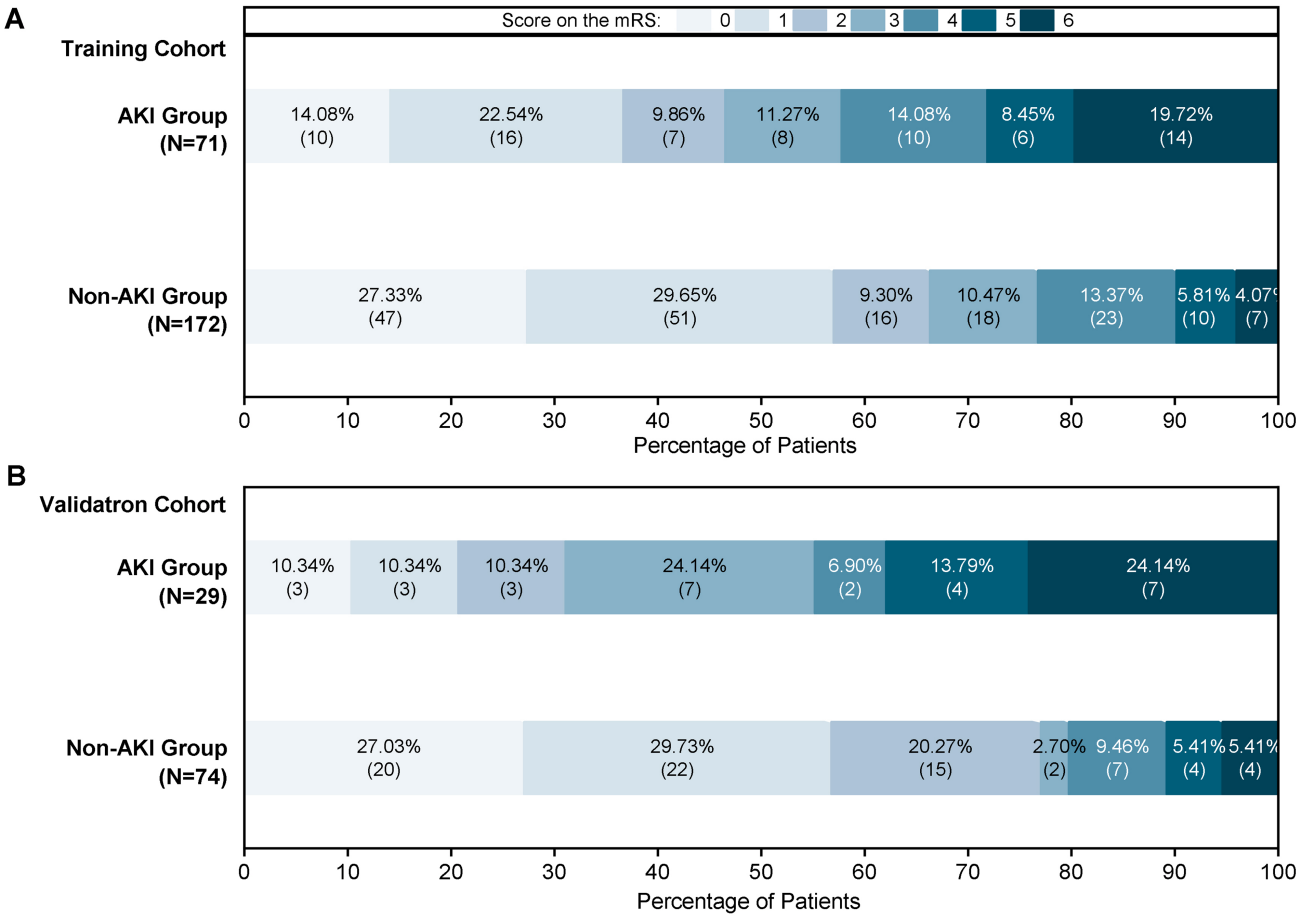
Proportions of AKI patients according to the 90-mRS results. **(A)** The 90-mRS training cohort. **(B)** The 90-mRS validation cohort. A total of 346 patients were included. The clinical outcomes of patients with AKI and non-AKI were followed up 3 months after EVT. The cut-off for functional dependence was set at an mRS of score >2.

This study enrolled 346 patients, who were divided into two groups: 246 without AKI (71.1%) and 100 with AKI (28.9%). To ensure reliability, all patients were randomly assigned to a training cohort (*n* = 243) and a validation cohort (*n* = 103) in a 7:3 ratio. Baseline characteristics are compared in Table 1 and Figure 2 illustrates the impact of AKI on neurological outcomes and 3-month mortality.

**Table 1:** A comparison of the baseline characteristics between the training cohort and validation cohort.

Variables		Training cohort			Validation cohort		
		Non-AKI group (*n* = 172)	AKI group (*n* = 71)	*p* value	Non-AKI group (*n* = 74)	AKI group (*n* = 29)	*p* value
Demographics							
Gender, male, *n* (%)		121 (70.35%)	55 (77.46%)	0.259	63 (85.14%)	15 (51.72%)	**<0.001**
Age, years, median (IQR)		60.14 ± 13.87	65.44 ± 14.29	**0.008**	58.96 ± 14.13	67.45 ± 14.60	**0.008**
Medical history							
Hypertension, *n* (%)		96 (55.81%)	57 (80.28%)	**<0.001**	40 (54.05%)	23 (79.31%)	**0.018**
Diabetes, *n* (%)		41 (23.84%)	24 (33.80%)	0.110	18 (24.32%)	11 (37.93%)	0.167
Atrial fibrillation, *n* (%)		25 (14.53%)	12 (16.90%)	0.641	14 (18.92%)	5 (17.24%)	0.843
Prior stroke, *n* (%)		21 (12.21%)	19 (26.76%)	0.005	14 (18.92%)	3 (10.34%)	0.448
Heart failure, *n* (%)		1 (0.58%)	2 (2.82%)	0.151	2 (2.70%)	2 (6.90%)	0.672
Smoking, *n* (%)		59 (34.30%)	31 (43.66%)	0.169	28 (37.84%)	8 (27.59%)	0.326
Alcohol drinking, *n* (%)		34 (19.77%)	16 (22.54%)	0.627	13 (17.57%)	5 (17.24%)	0.969
Clinical features							
Admission blood glucose, mmol/L, median (IQR)		6.50 (5.80–8.20)	7.30 (6–9.50)	**0.038**	8.19 (6.69–11.55)	8 (6.15–11.15)	0.058
Systolic pressure, mmHg, mean ± SD		149.98 ± 21.42	153.08 ± 24.45	0.326	152.23 ± 24.83	157.07 ± 20.94	0.356
Diastolic pressure, mmHg, mean ± SD		89.58 ± 13.20	89.13 ± 14.33	0.812	90.20 ± 13.43	90.17 ± 13.47	0.992
Admission NIHSS, score, median (IQR)		8.00 (3.00–13.00)	10.00 (6.00–15.00)	0.104	9.00 (5.00–13.00)	8.00 (5.00–14.00)	0.786
Admission GCS, score, median (IQR)		15.00 (13.00–15.00)	14.00 (12.00–15.00)	**0.016**	15.00 (13.00–15.00)	15.00 (13.00–15.00)	0.536
Admission mRS, 1–5 score, *n* (%)	1	27 (15.70%)	7 (9.86%)	0.218	7 (9.46%)	3 (10.34%)	0.848
	2	24 (13.95%)	6 (8.45%)		10 (13.51%)	5 (17.24%)	
	3	14 (8.14%)	10 (14.08%)		11 (14.86%)	4 (13.79%)	
	4	73 (42.44%)	31 (43.66%)		38 (51.35%)	11 (37.93%)	
	5	34 (19.77%)	17 (23.94%)		8 (10.81%)	6 (20.69%)	
Inspection results							
Scr, mg/dL, median (IQR)		72.60 (61.25–87.00)	91.00 (76.70–134)	**<0.001**	73.90 (61.83–83.23)	85.70 (65.00–111.50)	**0.017**
Cys C, mg/L, median (IQR)		1.06 (0.89–1.25)	1.31 (1.04–1.76)	**<0.001**	1.01 (0.87–1.16)	1.25 (1.05–1.48)	**<0.001**
ALB, mmol/L, mean ± SD		38.79 ± 3.68	36.43 ± 5.14	**<0.001**	38.83 ± 4.26	36.68 ± 5.20	**0.033**
Proteinuria, median (IQR)		41 (23.84%)	33 (46.48%)	**<0.001**	23 (31.08%)	13 (44.83%)	0.188
UA, umol/L, mean ± SD		329.54 ± 110.48	367.83 ± 114.82	**0.016**	313.65 ± 92.72	327.82 ± 88.50	0.482
CRP, mg/L, median (IQR)		3.51 (1.84–8.55)	4.80 (1.90–13.66)	0.234	3.20 (1.84–12.61)	5.92 (2.77–18.56)	**0.042**
WBC,10^9^/L, median (IQR)		9.92 (8.17–11.95)	10.08 (7.56–12.89)	0.540	9.39 (8.06–11.84)	10.73 (9.21–12.92)	0.050
Postoperative NLR, median (IQR)		4.91 (3.47–9.14)	6.79 (3.61–12.38)	0.075	5.25 (3.10–9.06)	6.50 (3.88–11.17)	0.129
Postoperative SII, median (IQR)		1,190.90 (751.14–2,132)	1,379.73 (860.81–2,475.61)	0.127	1,007.04 (681.72–1,882.86)	1,586 (850.20–2,167.49)	0.070
Postoperative PLR, median (IQR)		158 (112.60–234.00)	188.89 (133.52–282.86)	0.102	157.21 (100.70–254.99)	188.12 (119.24–247.17)	0.317
Postoperative SIRI, median (IQR)		1.97 (1.10–3.45)	2.08 (1.25–4.24)	0.137	1.77 (1.13–3.09)	2.36 (1.61–4.65)	0.050
Operations							
Operation time, hour, median (IQR)		1.78 (1.33–2.16)	1.83 (1.34–2.41)	0.180	1.53 (1.08–2.10)	2 (1.67–2.65)	**0.013**
Days in ICU, day, median (IQR)		1.78 (1.45–2.87)	3.96 (1.81–8.33)	**<0.001**	1.95 (1.52–2.78)	2.87 (1.57–7.98)	**0.038**
Duration of MV, hour, median (IQR)		0.67 (0.47–1.06)	1.67 (0.70–7.18)	**<0.001**	0.76 (0.50–1.61)	1.67 (0.75–1.67)	**0.002**
Postoperative complications				**0.021**			0.446
Intracranial hemorrhage, *n* (%)		14 (8.14%)	14 (19.72%)		7 (9.46%)	5 (17.24%)	
Gastrointestinal hemorrhage, *n* (%)		5 (2.91%)	4 (5.56%)		3 (4.05%)	2 (6.90%)	
Brain herniation, *n* (%)		4 (2.33%)	4 (5.56%)		0 (0.00%)	0 (0 0.00%)	
Prognosis				**<0.001**			**<0.001**
mRS 90 day, 1–6 score, *n* (%)	0	47 (27.33%)	10 (14.08%)		20 (20.03%)	3 (10.34%)	
	1	51 (29.65%)	16 (22.54%)		22 (29.73%)	3 (10.34%)	
	2	16 (9.30%)	7 (9.86%)		15 (20.27%)	3 (10.34%)	
	3	18 (10.47%)	8 (11.27%)		2 (2.70%)	7 (24.14%)	
	4	23 (13.37%)	10 (14.08%)		7 (9.46%)	2 (6.89%)	
	5	10 (5.81%)	6 (8.45%)		4 (5.41%)	4 (13.79%)	
	6	7 (4.07%)	14 (19.72%)		4 (5.41%)	7 (24.14%)	

IQR, Interquartile Range; NIHSS, National Institute of Health Stroke Scale; GCS, Glasgow Coma Scale; mRS, Modified Rankin Scale; Scr, Serum Creatinine; Cys C, Cystatin C; UA, Uric Acid; ALB, Albumin; WBC, White Blood Cell; CRP, C-reactive Protein; NLR = neutrophil count/lymphocyte count; SII = (neutrophil count × platelet count)/lymphocyte count; PLR = platelet count/lymphocyte count; SIRI = (neutrophil count × monocyte count)/lymphocyte count; ICU, Intensive Care Unit; MV, Mechanical Ventilation. Values with *P* < 0.05 are highlighted in bold.

### 3.3 Independent risk factors in the training cohort

**Figure 3:**
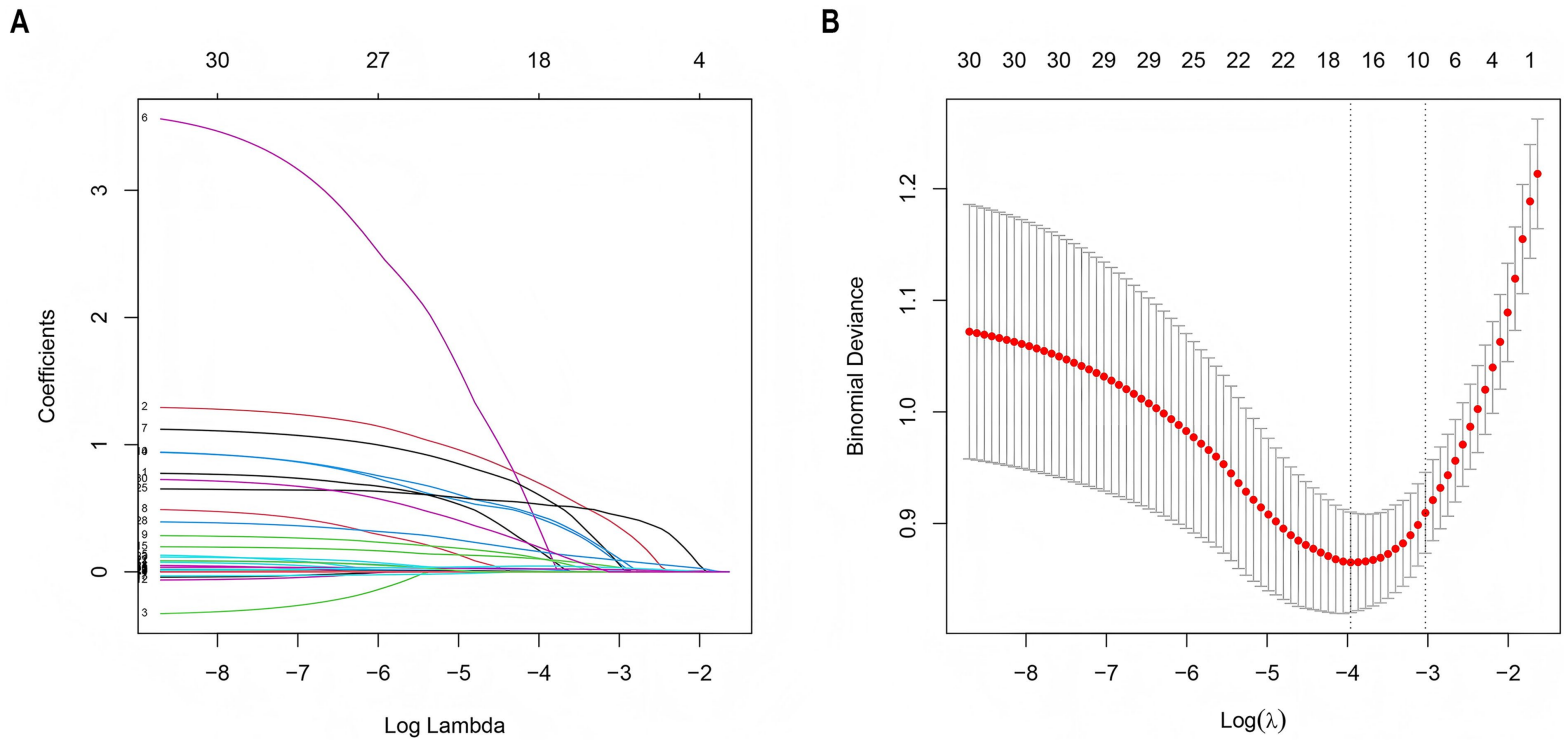
LASSO regression employed to further identify risk factors for AKI. **(A)** LASSO coefficient profiles of the 30 features. **(B)** Optimal parameter (*λ*) selection in the LASSO model used 2-fold cross-validation and minimum criteria. A coefficient profile plot was plotted against the log (*λ*) sequence. The 10 non-zero coefficients were chosen at the values selected using 10-fold cross-validation. LASSO, least absolute shrinkage and selection operator.

LASSO regression was employed to screen 30 variables for predictive ones with non-zero coefficients. (Figures 3A,B). By means of 10-fold cross-validation, the optimal *λ* value, which was the most appropriate for the model, was selected. While ensuring the goodness of fit, the fewest variables were included. Finally, lambda. Min (*λ* = 0.014) was chosen as the optimal *λ* value, and 10 predictive variables with non-zero coefficients were screened out: prior stroke, hypertension, smoking, proteinuria, admission blood glucose, SIRI, Scr, cystatin C, days in ICU, and duration of mechanical ventilation.

### 3.4 Prediction model development

**Figure 4:**
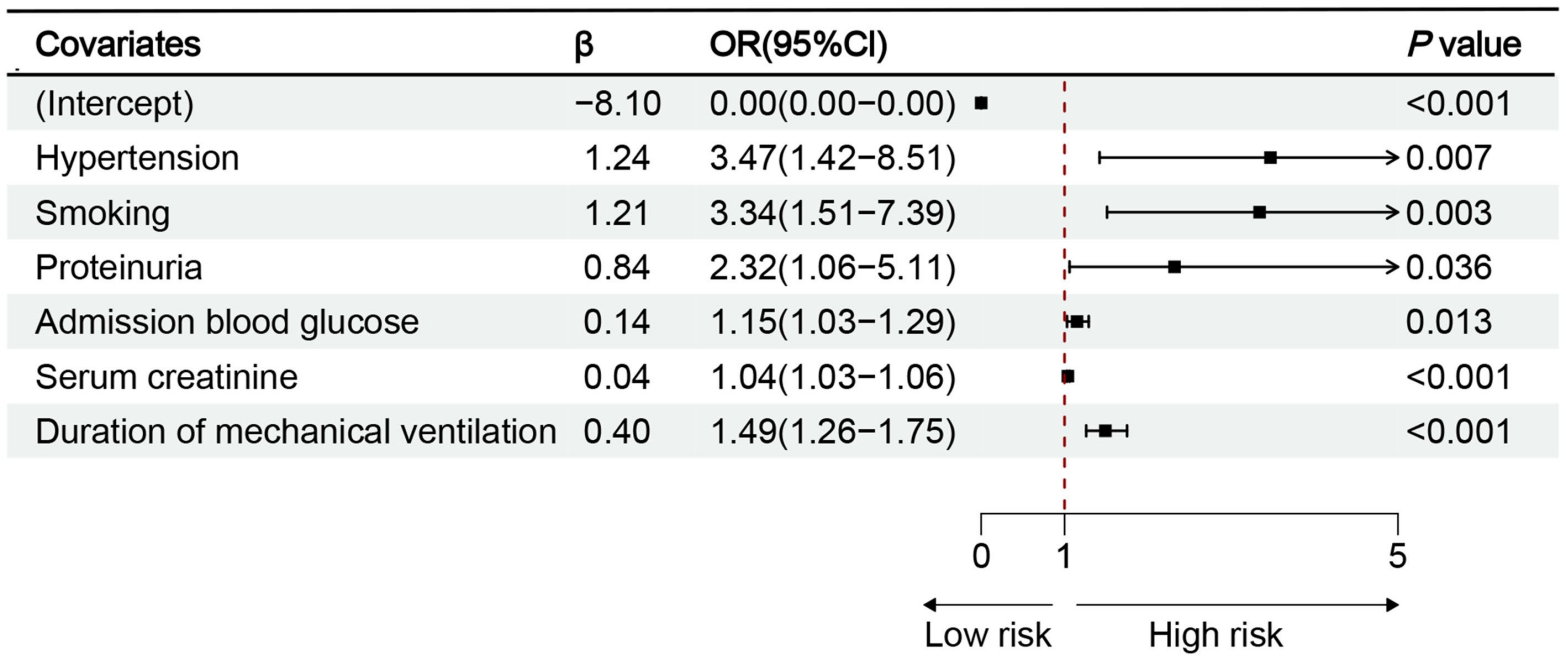
Results of multinomial logistic regression analysis of AKI after EVT. OR, odds ratio; CI, confidence interval; *p* < 0.05.

**Figure 5:**
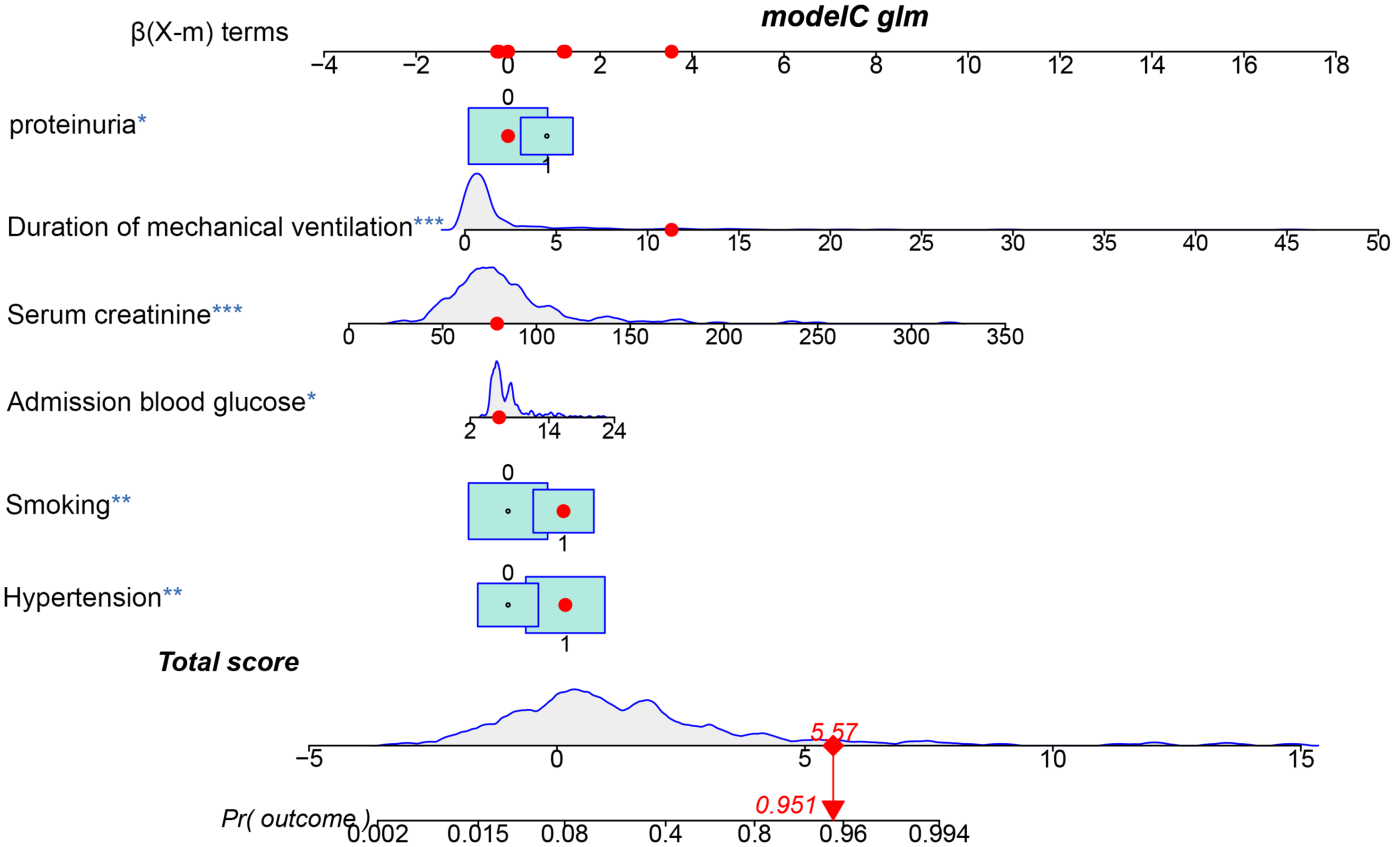
Nomogram predicting the probability of AKI in patients after EVT of the training cohort. The dots represent the prediction results of patients.

The 10 variables obtained through screening by LASSO were included in the multinomial logistic regression. The multinomial logistic regression analysis using the stepwise backward regression method showed that hypertension (OR 3.47, 95%CI 1.42–8.51, *p* = 0.007), smoking (OR 3.34, 95%CI 1.51–7.39, *p* < 0.003), proteinuria (OR 2.32, 95%CI 1.06–5.11, *p* = 0.036), admission blood glucose (OR 1.15, 95%CI 1.03–1.29, *p* = 0.013), Scr (OR 1.04, 95%CI 1.03–1.06, *p* < 0.001), and duration of mechanical ventilation (OR 1.49, 95%CI 1.26–1.75, *p* < 0.001) were still independent influencing factors for the occurrence of AKI (*p* < 0.05) (Figure 4). According to the results, a nomogram model for predicting the risk of AKI after EVT was developed using these six risk factors: hypertension, smoking, proteinuria, admission blood glucose, Scr, and duration of mechanical ventilation (Figure 5).

### 3.5 ROC validation of predictive model

**Figure 6:**
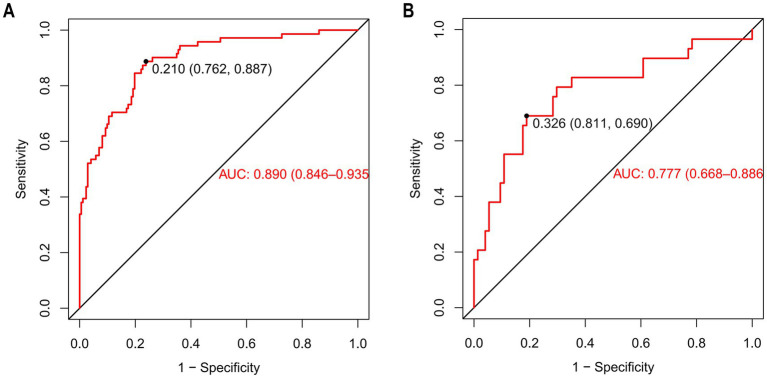
The ROC of nomogram for predicting patients with AKI. **(A)** (Training cohort), the ROC curve has an AUC of 0.890 (95% CI, 0.846–0.935); the marked Youden index is 0.210, corresponding to a sensitivity of 0.762 and a specificity of 0.887. **(B)** (Validation cohort), the AUC of the ROC curve is 0.777 (95% CI, 0.668–0.886), with a Youden index of 0.326 that corresponds to a sensitivity of 0.811 and a specificity of 0.690. ROC curves are used to evaluate the model’s ability to distinguish between patients with AKI and those without AKI. The closer the AUC is to 1, the better the model’s predictive performance.

The discriminatory ability of the nomogram was assessed by computing the area under the ROC curve, which is denoted as AUC. The results showed that the AUC of the training cohort was 0.890 [95%CI (0.846–0.935)] (Figure 6A). The cut-off value associated with the maximum Youden index was 0.210. At this value, the sensitivity reached 0.887 and the specificity was 0.762. The model of the training cohort was applied to the validation cohort for verification. The AUC of the validation cohort was 0.777 [95%CI (0.668–0.886)] (Figure 6B). The maximum Youden index corresponded to a cut-off value of 0.326, which had a sensitivity of 0.690 and a specificity of 0.811.

### 3.6 Measurement of model performance

The bootstrap optimism-corrected measure was used to adjust for model overfitting. A model was first developed on the entire original dataset and its apparent performance was recorded. One thousand bootstrap samples were then generated by random sampling with replacement from the original dataset. For each sample, a logistic model was constructed and its performance was evaluated on both the bootstrap sample and the original dataset. Optimism was calculated as the difference between these two performances for each sample. After 1,000 iterations, the average optimism was obtained and subtracted from the apparent performance of the original model to yield the optimism-corrected performance. The model performance was also evaluated by 10-fold cross validation. The dataset was randomly divided into 10 equally sized subsets. In each iteration, one subset was retained as the test set while the remaining 9 subsets were combined to form the training set. This process was repeated 10 times, ensuring that each subset served as the test set exactly once. Model performance metrics were then averaged across the 10 folds, providing a robust estimate of generalization performance and mitigating the risk of overfitting (Table 2).

**Table 2:** Results of cross-validation and bootstrap analysis.

Measures	Fold cross-validation (CI 95%)	Bootstrap optimism-corrected (CI 95%)
Auroc	0.859 (0.787–0.930)	0.851 (0.811–0.897)
Sensitivity	0.936 (0.909–0.963)	0.929 (0.895–0.960)
Specificity	0.503 (0.389–0.617)	0.526 (0.430–0.631)
Accuracy	0.811 (0.768–0.855)	0.812 (0.772–0.853)
Precision	0.822 (0.766–0.879)	0.828 (0.788–0.870)
Recall	0.936 (0.909–0.963)	0.929 (0.895–0.960)

OR, odds ratio; CI, confidence interval.

### 3.7 Performance evaluation of predictive model

**Figure 7:**
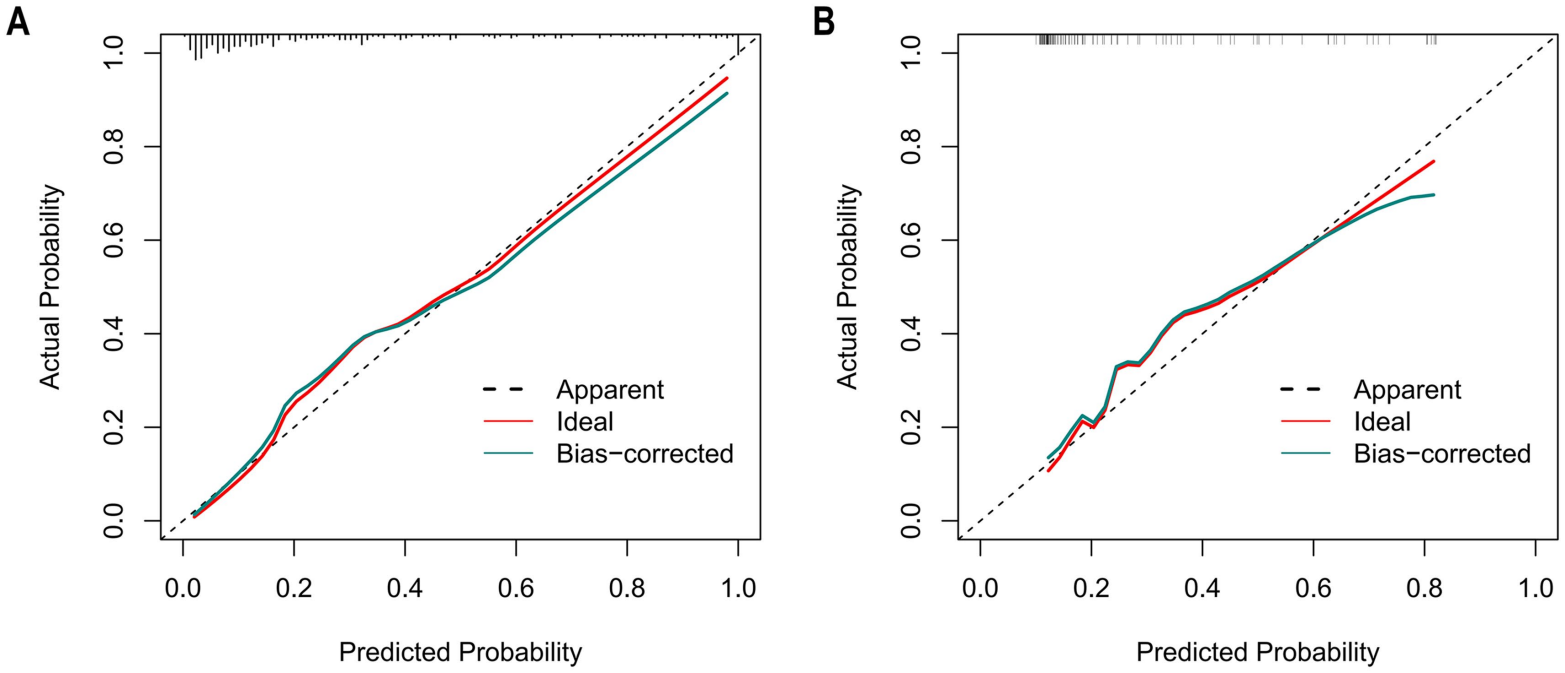
The calibration curves of the nomogram for predicting in patients with AKI. **(A)** The calibration curves of the nomogram in the training cohort; **(B)** The calibration curves of the nomogram in the validation cohort. In these plots, the *X*-axis denotes the predicted AKI probability, and the *Y*-axis denotes the actual AKI incidence. The 45° dashed line signifies ideal prediction (a perfect match between predicted and actual probabilities). The “Apparent” dashed line shows the model’s uncorrected predictions, the “Ideal” red line is the benchmark, and the “Bias-corrected” green line is after bias adjustment. Calibration curves assess how well predicted and actual probabilities align; closer alignment to the 45° line means higher accuracy. After bias correction, the nomogram’s predictions in both cohorts closely matched the ideal, demonstrating good accuracy in predicting the AKI occurrence probability.

**Figure 8:**
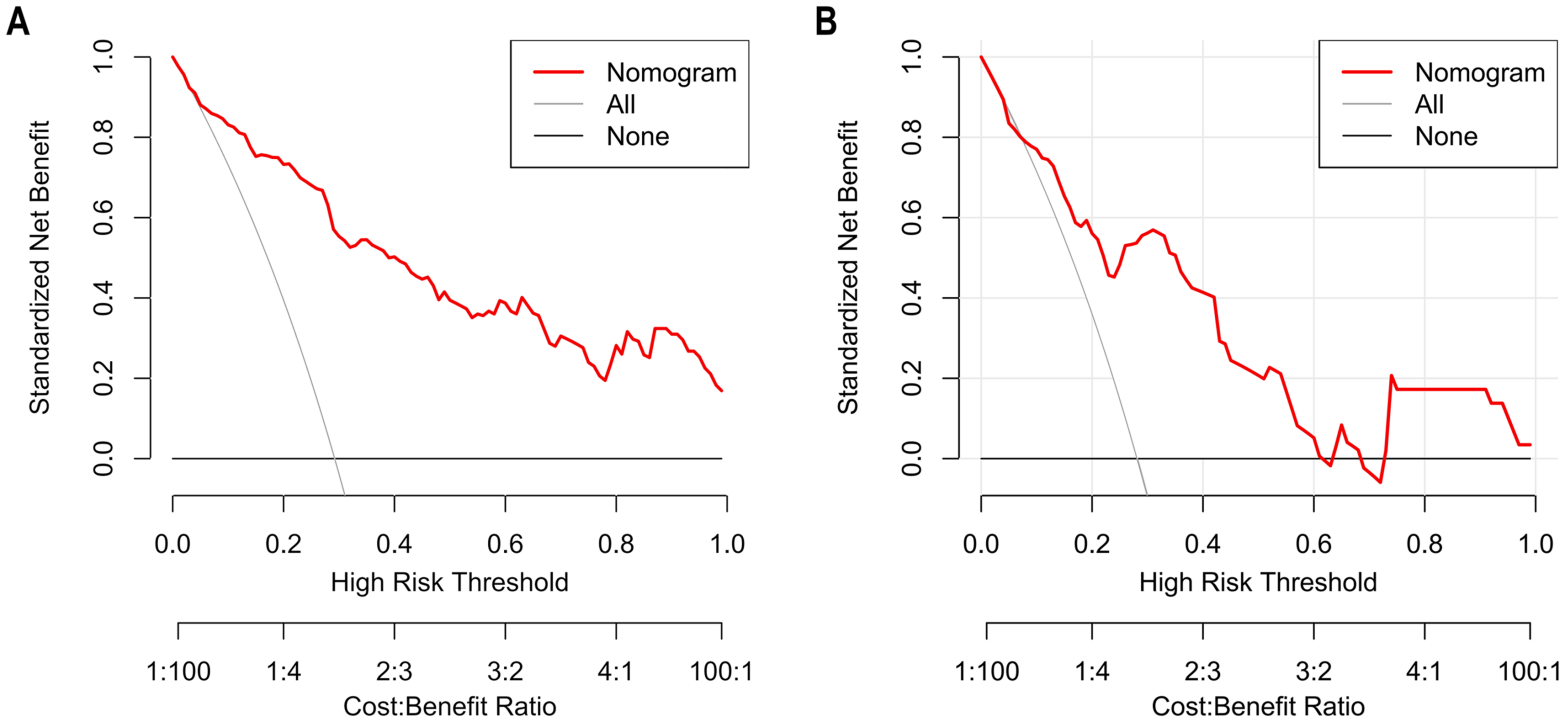
The calibration curves of the nomogram for predicting in patients with AKI **(A)** The calibration curves of the nomogram in the training cohort; **(B)**. The calibration curves of the nomogram in the validation cohort. In these plots, the *Y*-axis represents standardized net benefit and the *X*-axis represents high-risk threshold probability, and the Y-axis denotes the actual AKI incidence. The 45° dashed line signifies ideal prediction (a perfect match between predicted and actual probabilities). The “Apparent” dashed line shows the model’s uncorrected predictions, the “Ideal” red line is the benchmark, and the “Bias-corrected” green line is after bias adjustment. Calibration curves assess how well predicted and actual probabilities align; closer alignment to the 45° line means higher accuracy. After bias correction, the nomogram’s predictions in both cohorts closely matched the ideal, demonstrating good accuracy in predicting the AKI occurrence probability.

**Figure 9:**
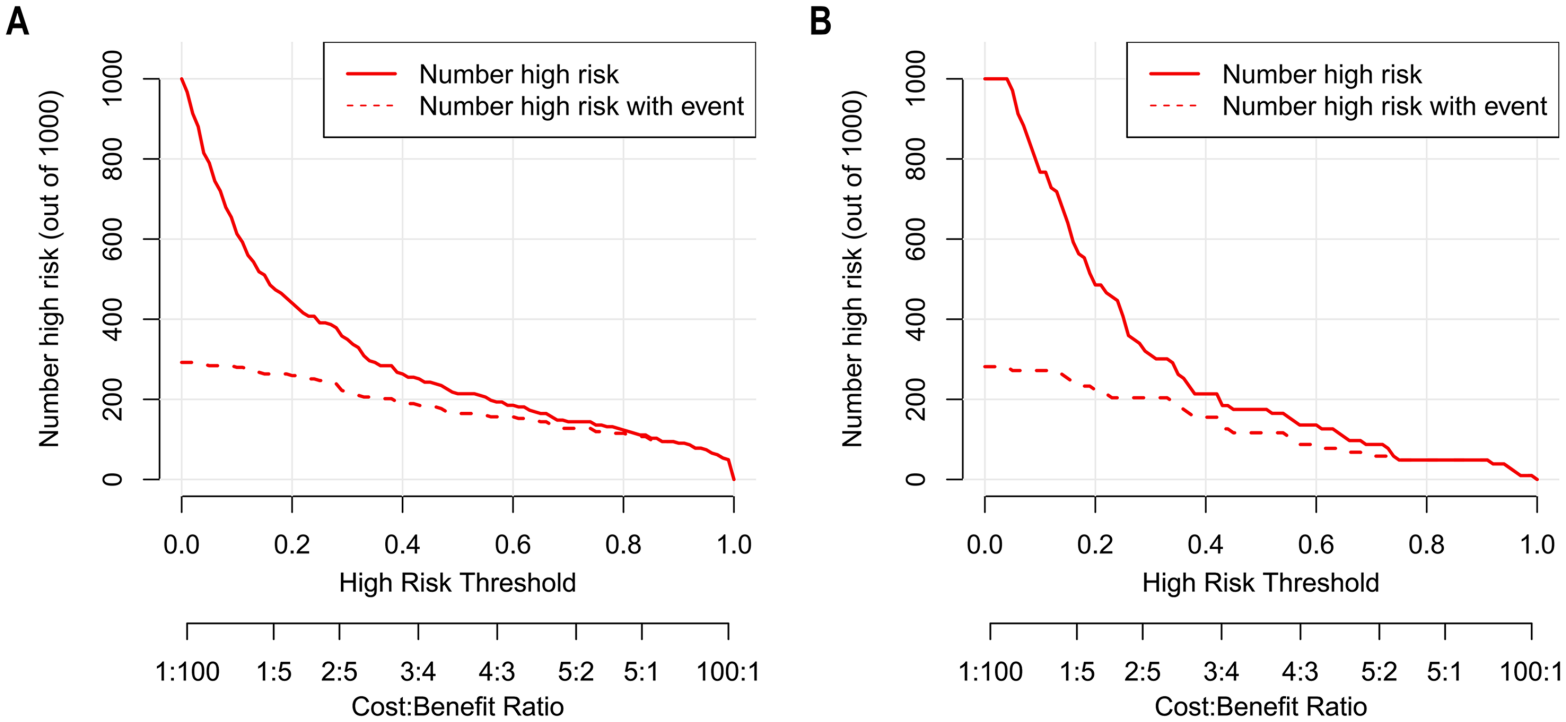
The CIC of nomogram for predicting in patients with AKI. **(A)** The CIC of nomogram in the training cohort; **(B)** The CIC of the nomogram in the validation cohort. The *x*-axis shows the risk threshold, and the *y*-axis represents the number of people at risk. The solid red line (the number of high-risk individuals) indicates the number of people classified as positive by the model at each threshold probability; the dashed red line (the number of high-risk individuals with outcomes) indicates the number of true positives at each threshold probability. CIC reflects a model’s clinical value in identifying high-risk populations. A model has high net benefit if it differentiates well (gap between the two red lines) and identifies reasonable true positives with rational high-risk population size. As shown, the nomogram stably identified true AKI cases in both cohorts with reasonable high-risk size, verifying its clinical utility.

The Hosmer–Lemeshow goodness-of-fit test was utilized to assess the calibration of the predictive model. As depicted in the calibration curve (Figures 7A,B), a robust correlation was demonstrated between the predicted probabilities and the actual incidence of AKI in both the validation cohort and training cohort. We conducted the DCA to evaluate the clinical applicability of our nomogram. The DCA curve indicated that within a reasonable range of threshold probabilities, the nomogram model could provide clinical net benefit (Figures 8A,B) for patients in both the training cohort and validation cohort. On this basis, we plotted the CIC (Figures 9A,B) to assess the clinical impact of the model. The CIC shows a high degree of agreement between the number of positive events and the actual incidence of AKI.

## 4 Discussion

AIS is one of the diseases that cause the highest disability and mortality rates in China (24). Despite the breakthroughs in EVT technology, some AIS-LVO patients still fail to achieve a good long-term prognosis after EVT treatment and may even face severe disability or death (1, 25). AKI is a relatively common complication in AIS-LVO patients after EVT (26). Our results showed that EVT led to AKI in 28.90% of patients with AIS-LVO. To further explore the impact of AKI on patient outcomes, we conducted a systematic follow-up 3 months after EVT. Data comparison revealed that patients with AKI had a significantly higher proportion of poor functional outcomes at 90 days in both cohorts. This result indicated that AKI has a significant negative impact on neurological recovery in patients with AIS-LVO. It not only delays the recovery process of neurological function, but also significantly increases the risk of permanent neurological dysfunction. In conclusion, it is of great significance to identify the potential factors for the occurrence of AKI in patients with AIS-LVO at an early stage. This not only helps clinicians take more effective preventive measures in advance to reduce the incidence of AKI but also enables them to timely adjust the treatment plan in the early stage of the disease, thus reducing mortality and improving the quality of life of patients.

Notably, the nomogram constructed in this study, combined with the clinical net benefit confirmed by DCA, can provide precise guidance for the post-EVT care of patients with AIS-LVO. In fluid management, stratified protocols based on the nomogram’s AKI risk stratification are feasible: high-risk patients may receive “restrictive precision fluid replacement” to avoid increasing renal filtration burden, while moderate-to-low-risk patients maintain conventional fluid replacement to ensure renal perfusion, addressing volume imbalance from empirical fluid administration. For contrast medium use, the nomogram’s reliable predictive performance aids decision-making: high-risk patients (e.g., with proteinuria or elevated admission blood glucose) are prioritized for isotonic, low-toxicity contrast media, with pre-procedural renal function assessment and optional sodium bicarbonate for nephrotoxicity prevention; low-risk patients use conventional contrast media, balancing diagnostic efficacy and renal safety per DCA’s “risk–benefit” principle. In other prevention strategies, the nomogram enables risk-targeted interventions: high-risk patients receive optimized respiratory support (based on variables like “mechanical ventilation duration”) to shorten ventilation time and enhanced post-procedural serum creatinine monitoring; modifiable factors (hypertension, smoking) are managed via perioperative blood pressure control and smoking cessation guidance. These measures effectively translate DCA-confirmed net benefit into clinical practice improvements, reducing AKI risk.

Multinomial Logistic regression analysis results indicated that hypertension, smoking, proteinuria, admission blood glucose, Scr, and duration of mechanical ventilation were all identified as independent factors influencing the occurrence of AKI. Prior research has concentrated on the association between smoking and the risk of stroke. However, there is a lack of studies exploring the influence of smoking on post-stroke renal injury (27). Based on the above, this study speculated that long-term exposure to nicotine, through the oxidative stress induced by reperfusion, could directly trigger AKI or aggravate its symptoms. Additionally, it would also increase the production of mitochondrial reactive oxygen species (ROS) in renal proximal tubular cells (RPTCs) (28). For patients with hypertension, long-term elevation of blood pressure has been proved to lead to thickening of renal vessel wall and narrowing of renal lumen, which causes changes in renal hemodynamics and affects renal perfusion and filtration function, making these patients more susceptible to renal injury after stroke (29). In addition, hypertension can affect the metabolism and excretion of drugs and increase nephrotoxicity (30). Therefore, it is necessary to evaluate hypertension and renal function before surgical intervention. Moreover, blood pressure should be enhanced after admission to reduce the risk of renal impairment.

Scr is an endogenous creatinine produced by muscle metabolism. It has a small molecule and is excreted through the glomerulus with urine. A rise in its level serves as an indication of compromised kidney function. The study by Wong has found that patients with elevated baseline Scr levels are at a higher risk of developing AKI. Additionally, these patients are more prone to the progression of AKI, which ultimately leads to decreased survival (31). The conclusion of the present study is basically consistent with this. Proteinuria is an important marker of renal prognosis, independent of other risk factors, and a predictor of progressive renal impairment as well as mortality (32, 33). For individual patients, the risk of renal impairment and end-stage renal failure increases as proteinuria levels rise (34). Existing studies have shown that proteinuria is a definite risk factor for cerebrovascular diseases and will increase the risk of poor functional outcomes. Other studies have found an independent association between proteinuria and the risk of developing AKI. Rocco’s study found that patients with proteinuria had a higher risk of postoperative AKI, as well as an increased risk of kidney disease progression (35). For patients undergoing EVT, proteinuria can be a sign of systemic endothelial dysfunction. This condition results in a decreased ability of the kidney to tolerate the hemodynamic changes that occur during surgery, ultimately leading to postoperative AKI (36, 37). Urinary protein has inherent profibrotic properties, which can cause chronic tubulointerstitial injury, thereby attenuating the ability of the proximal tubules to reabsorb filtered albumin (38, 39). For a long time, there have been few studies on proteinuria as an early risk factor for AKI in patients after EVT for AIS-LVO. The results of this study have filled some of the gaps in this area.

Admission blood glucose is an index that is extremely easy to obtain. The detection process does not require professional technical knowledge or special equipment and can be completed simply by fingertip blood collection. Our study shows that blood glucose levels at admission can be used as a risk factor for predicting the development of AKI after EVT. Multiple previous studies have shown that elevated blood glucose on admission is associated with an increased risk of AKI (40, 41). Hyperglycemia can increase ischemic brain injury by increasing tissue lactate formation and impairing normal phosphorus metabolism (42). At the same time, it can also enhance insulin resistance and exacerbate the inflammatory and oxidative stress responses. Since these factors are all involved in the development process of AKI, the hyperglycemic state may promote the occurrence of AKI. This suggests that in addition to paying attention to the patient’s history of diabetes, more attention should be paid to the blood glucose level at admission, and it should be used as an important reference index to evaluate the risk of AKI after EVT.

Notably, our research has found that the duration of mechanical ventilation may be a predictor of AKI risk. Most patients who have received EVT will be transferred to the ICU for observation. In the ICU, mechanical ventilation plays an important role as a common life support measure. However, it cannot be ignored that mechanical ventilation can double the risk of patients developing AKI (43). Numerous studies have clearly shown that the occurrence of AKI during mechanical ventilation is significantly correlated with the length of stay of patients in the ICU and ventilator time of patients (44). The kidney has special physiological structure, rich blood supply and high blood perfusion requirements, and is extremely sensitive to ischemia and hypoxia. Compared with other organs, it are more likely to prone to dysfunction in the face of similar ischemia and hypoxia. Based on this, some scholars have proposed the concept of ventilator-induced kidney injury (VIKI) (8). Mechanical ventilation can cause a decrease in the glomerular filtration rate, which significantly increases the risk of AKI (43, 45, 46). In addition, systemic inflammatory response and oxidative stress caused by AKI aggravate acute lung injury (ALI), increase the difficulty of weaning from the ventilator, and ultimately lead to prolonged mechanical ventilation (8). In conclusion, when improving cerebral blood flow reperfusion after EVT, it is necessary to pay attention to the management of mechanical ventilation. We should strictly control the infection, make every effort to shorten the duration of mechanical ventilation, and complete the extubation operation as early as possible. This not only helps to reduce the risk of AKI, but also significantly improves the prognosis of patients.

At present, precisely predicting the risk of AKI in patients with AIS-LVO after EVT remains a significant challenge in the medical field. In this study, we constructed a nomogram for predicting the risk factors of AKI in patients treated with EVT. ROC curve analysis showed that the AUC of the model for predicting AKI in EVT patients reached 0.890, which fully indicated that the prediction model constructed had good predictive performance. Based on this, clinicians can implement precise and targeted treatment or intervention measures for patients with relevant characteristics according to this model, effectively reducing the risk of AKI in patients, thus significantly improving the prognosis of patients and improving their quality of life.

## 5 Conclusion

In our study, 28.90% of AIS-LVO patients were diagnosed with AKI after undergoing EVT. We found that hypertension, smoking, admission blood glucose, Scr, proteinuria, and the duration of mechanical ventilation were significantly correlated with the occurrence of AKI. Based on this finding, we developed a nomogram prediction model. This model is specifically designed to provide visualized accurate predictions of the risk of AKI in patients with AIS-LVO after undergoing EVT. This breakthrough holds significant clinical implications. On the one hand, doctors can formulate personalized intervention measures targeting modifiable risk factors to effectively reduce the risk of AKI. On the other hand, for the identified high-risk patients, renal function can be monitored and intervened in a timely manner at an early stage. This helps to delay the progression of AKI and avoid the development of end-stage renal disease in patients to the greatest extent.

## 6 Limitations

This study has certain limitations. (1) In this study, patients with AIS-LVO required loop diuretics to control cerebral edema within 24 h after EVT. Due to the difficulty in distinguishing iatrogenic oliguria induced by diuretics from pathological oliguria caused by AKI, the urine output criterion specified in the 2012 KDIGO criteria was not included in AKI diagnosis, which may lead to an underestimation of the actual disease burden. The incidence of AKI in this study was 28.9%, which is highly consistent with the 25–30% AKI incidence diagnosed “solely based on serum creatinine” in the study by Rui Du et al. (47). Bias was reduced through strict inclusion criteria and hemodynamic monitoring. Furthermore, the current model already has a solid foundation in predictive performance (AUC = 0.890 in the training cohort and AUC = 0.777 in the validation cohort). For subsequent research, we plan to conduct a multicenter prospective study and incorporate the corrected urine output index into the model to optimize its performance. (2) Because all patients had already received contrast medium during EVT, and considering the retrospective characteristic of this study, the contrast was not recorded in our electronic medical record system. This lack of data directly prevented us from including contrast medium dose in the study analysis. In future studies, we should set the contrast medium dose as a core variable to optimize the data acquisition process and ensure accuracy of the dose information. (3) In this study, the validation work has not yet included verification using an external independent dataset, which will also be a key direction for improvement in our subsequent research. To ensure the rigor of model validation, we have supplemented two validation methods, namely the bootstrap optimism correction and the 10-fold cross-validation, to further control the risk of overfitting and enhance the robustness of the validation results. These two methods have effectively strengthened the validation process of the model’s performance and ensured the reliability of the validation results.

## Data Availability

The raw data supporting the conclusions of this article will be made available by the authors without undue reservation.

## References

[1] Warner JJ Harrington RA Sacco RL Elkind M. Guidelines for the early management of patients with acute ischemic stroke: 2019 update to the 2018 guidelines for the early management of acute ischemic stroke. Stroke. (2019) 50:3331–2. doi: 10.1161/STROKEAHA.119.027708, PMID: 31662117

[2] Goyal M Menon BK van Zwam WH Dippel DW Mitchell PJ Demchuk AM. Endovascular thrombectomy after large-vessel ischaemic stroke: a meta-analysis of individual patient data from five randomised trials. Lancet. (2016) 387:1723–31. doi: 10.1016/S0140-6736(16)00163-X, PMID: 26898852

[3] Goyal M Demchuk AM Menon BK Eesa M Rempel JL Thornton J. Randomized assessment of rapid endovascular treatment of ischemic stroke. N Engl J Med. (2015) 372:1019–30. doi: 10.1056/NEJMoa1414905, PMID: 25671798

[4] Pilgram-Pastor SM Piechowiak EI Dobrocky T Kaesmacher J Den Hollander J Gralla J. Stroke thrombectomy complication management. J Neurointerv Surg. (2021) 13:912–7. doi: 10.1136/neurintsurg-2021-017349, PMID: 34158401 PMC8458081

[5] Diprose WK Sutherland LJ Wang M Barber PA. Contrast-associated acute kidney injury in endovascular thrombectomy patients with and without baseline renal impairment. Stroke. (2019) 50:3527–31. doi: 10.1161/STROKEAHA.119.026738, PMID: 31587663

[6] Yoo J Hong JH Lee SJ Kim YW Hong JM Kim CH. Acute kidney injury after endovascular treatment in patients with acute ischemic stroke. J Clin Med. (2020) 9:9. doi: 10.3390/jcm9051471, PMID: 32422921 PMC7291207

[7] Fandler-Hofler S Odler B Kneihsl M Wunsch G Haidegger M Poltrum B. Acute and chronic kidney dysfunction and outcome after stroke thrombectomy. Transl Stroke Res. (2021) 12:791–8. doi: 10.1007/s12975-020-00881-2, PMID: 33398648 PMC8421282

[8] Wu GC Peng CK Liao WI Pao HP Huang KL Chu SJ. Melatonin receptor agonist protects against acute lung injury induced by ventilator through up-regulation of IL-10 production. Respir Res. (2020) 21:65. doi: 10.1186/s12931-020-1325-2, PMID: 32143642 PMC7059294

[9] Chertow GM Burdick E Honour M Bonventre JV Bates DW. Acute kidney injury, mortality, length of stay, and costs in hospitalized patients. J Am Soc Nephrol. (2005) 16:3365–70. doi: 10.1681/ASN.2004090740, PMID: 16177006

[10] Covic A Schiller A Mardare NG Petrica L Petrica M Mihaescu A. The impact of acute kidney injury on short-term survival in an eastern european population with stroke. Nephrol Dial Transplant. (2008) 23:2228–34. doi: 10.1093/ndt/gfm591, PMID: 17989102

[11] Ferenbach DA Bonventre JV. Mechanisms of maladaptive repair after AKI leading to accelerated kidney ageing and CKD. Nat Rev Nephrol. (2015) 11:264–76. doi: 10.1038/nrneph.2015.3, PMID: 25643664 PMC4412815

[12] Palevsky PM Liu KD Brophy PD Chawla LS Parikh CR Thakar CV. KDOQI US commentary on the 2012 KDIGO clinical practice guideline for acute kidney injury. Am J Kidney Dis. (2013) 61:649–72. doi: 10.1053/j.ajkd.2013.02.349, PMID: 23499048

[13] Levey AS James MT. Acute kidney injury. Ann Intern Med. (2017) 167:ITC66-80. doi: 10.7326/AITC201711070, PMID: 29114754

[14] Al-Naimi MS Rasheed HA Hussien NR Al-Kuraishy HM Al-Gareeb AI. Nephrotoxicity: role and significance of renal biomarkers in the early detection of acute renal injury. J Adv Pharm Technol Res. (2019) 10:95–9. doi: 10.4103/japtr.JAPTR_336_18, PMID: 31334089 PMC6621352

[15] Lacquaniti A Ceresa F Campo S Smeriglio A Trombetta D Patane F. Surgical aortic valve replacement and renal dysfunction: from acute kidney injury to chronic disease. J Clin Med. (2024) 13:13. doi: 10.3390/jcm13102933, PMID: 38792474 PMC11122348

[16] Privratsky JR Krishnamoorthy V Raghunathan K Ohnuma T Rasouli MR Long TE. Postoperative acute kidney injury is associated with progression of chronic kidney disease independent of severity. Anesth Analg. (2022) 134:49–58. doi: 10.1213/ANE.0000000000005702, PMID: 34908546

[17] Shoji S Kuno T Kohsaka S Amiya E Asleh R Alvarez P. Incidence and long-term outcome of heart transplantation patients who develop postoperative renal failure requiring dialysis. J Heart Lung Transplant. (2022) 41:356–64. doi: 10.1016/j.healun.2021.11.017, PMID: 34953720

[18] Kayar SR Hoppeler H Mermod L Weibel ER. Mitochondrial size and shape in equine skeletal muscle: a three-dimensional reconstruction study. Anat Rec. (1988) 222:333–9. doi: 10.1002/ar.1092220405, PMID: 3228204

[19] Bhatraju PK Zelnick LR Chinchilli VM Moledina DG Coca SG Parikh CR. Association between early recovery of kidney function after acute kidney injury and long-term clinical outcomes. JAMA Netw Open. (2020) 3:e202682. doi: 10.1001/jamanetworkopen.2020.2682, PMID: 32282046 PMC7154800

[20] Drubel K Marahrens B Ritter O Patschan D. Kidney-related outcome in cardiorenal syndrome type 3. Int J Nephrol. (2022) 2022:4895434. doi: 10.1155/2022/4895434, PMID: 35178254 PMC8844349

[21] Wang T Jin X Yang P Li S Zhang Q Shao C. A clinical and computed tomography-based nomogram to predict the outcome in patients with anterior circulation large vessel occlusion after endovascular mechanical thrombectomy. Jpn J Radiol. (2024) 42:973–82. doi: 10.1007/s11604-024-01583-7, PMID: 38700623

[22] Wang Y Yuan X Kang Y Yu L Chen W Fan G. Clinical predictors of prognosis in stroke patients after endovascular therapy. Sci Rep. (2024) 14:667. doi: 10.1038/s41598-024-51356-5, PMID: 38182739 PMC10770320

[23] Arora S Agrawal A Vishnu VY Singh MB Goyal V Srivastava P. Navigating the nexus: acute kidney injury in acute stroke - a prospective cohort study. Ann Indian Acad Neurol. (2024) 27:384–92. doi: 10.4103/aian.aian_177_24, PMID: 39172071 PMC11418761

[24] Fan J Li X Yu X Liu Z Jiang Y Fang Y. Global burden, risk factor analysis, and prediction study of ischemic stroke, 1990-2030. Neurology. (2023) 101:e137–50. doi: 10.1212/WNL.0000000000207387, PMID: 37197995 PMC10351546

[25] Chen H Lee JS Michel P Yan B Chaturvedi S. Endovascular stroke thrombectomy for patients with large ischemic core: a review. JAMA Neurol. (2024) 81:1085–93. doi: 10.1001/jamaneurol.2024.2500, PMID: 39133467

[26] Husain-Syed F Takeuchi T Neyra JA Ramirez-Guerrero G Rosner MH Ronco C. Acute kidney injury in neurocritical care. Crit Care. (2023) 27:341. doi: 10.1186/s13054-023-04632-1, PMID: 37661277 PMC10475203

[27] Harshfield EL Georgakis MK Malik R Dichgans M Markus HS. Modifiable lifestyle factors and risk of stroke: a mendelian randomization analysis. Stroke. (2021) 52:931–6. doi: 10.1161/STROKEAHA.120.031710, PMID: 33535786 PMC7903981

[28] Arany I Clark J Reed DK Juncos LA. Chronic nicotine exposure augments renal oxidative stress and injury through transcriptional activation of p66shc. Nephrol Dial Transplant. (2013) 28:1417–25. doi: 10.1093/ndt/gfs596, PMID: 23328708 PMC3685305

[29] Georgianos PI Agarwal R. Hypertension in chronic kidney disease-treatment standard 2023. Nephrol Dial Transplant. (2023) 38:2694–703. doi: 10.1093/ndt/gfad118, PMID: 37355779 PMC10689140

[30] Barbieri L Verdoia M Suryapranata H De Luca G. Impact of vascular access on the development of contrast induced nephropathy in patients undergoing coronary angiography and/or percutaneous coronary intervention. Int J Cardiol. (2019) 275:48–52. doi: 10.1016/j.ijcard.2018.08.026, PMID: 30274753

[31] Wong F O’Leary JG Reddy KR Garcia-Tsao G Fallon MB Biggins SW. Acute kidney injury in cirrhosis: baseline serum creatinine predicts patient outcomes. Am J Gastroenterol. (2017) 112:1103–10. doi: 10.1038/ajg.2017.122, PMID: 28440305

[32] Jun J Park K Lee HS Lee KW Lee JE Park JB. Clinical relevance of postoperative proteinuria for prediction of early renal outcomes after kidney transplantation. Kidney Res Clin Pract. (2022) 41:707–16. doi: 10.23876/j.krcp.21.246, PMID: 35977905 PMC9731780

[33] Cravedi P Remuzzi G. Pathophysiology of proteinuria and its value as an outcome measure in chronic kidney disease. Br J Clin Pharmacol. (2013) 76:516–23. doi: 10.1111/bcp.12104, PMID: 23441592 PMC3791975

[34] Su L Li Y Chen R Zhang X Cao Y Luo F. Epidemiology and outcomes of post-AKI proteinuria. Clin Kidney J. (2023) 16:2262–70. doi: 10.1093/ckj/sfad129, PMID: 37915920 PMC10616502

[35] Flammia RS Tufano A Proietti F Gerolimetto C Nunzio CDE Franco G. Renal surgery for kidney cancer: is preoperative proteinuria a predictor of functional and survival outcomes after surgery? A systematic review of the literature. Minerva Urol Nefrol. (2022) 74:255–64. doi: 10.23736/S2724-6051.21.04308-1, PMID: 34156198

[36] Chang CY Chien YJ Kao MC Lin HY Chen YL Wu MY. Pre-operative proteinuria, postoperative acute kidney injury and mortality: a systematic review and meta-analysis. Eur J Anaesthesiol. (2021) 38:702–14. doi: 10.1097/EJA.0000000000001542, PMID: 34101638

[37] Wahl TS Graham LA Morris MS Richman JS Hollis RH Jones CE. Association between preoperative proteinuria and postoperative acute kidney injury and readmission. JAMA Surg. (2018) 153:e182009. doi: 10.1001/jamasurg.2018.2009, PMID: 29971429 PMC6233648

[38] Kwiatkowska E Domanski L Dziedziejko V Kajdy A Stefanska K Kwiatkowski S. The mechanism of drug nephrotoxicity and the methods for preventing kidney damage. Int J Mol Sci. (2021) 22:22. doi: 10.3390/ijms22116109, PMID: 34204029 PMC8201165

[39] D’Amico G Bazzi C. Pathophysiology of proteinuria. Kidney Int. (2003) 63:809–25. doi: 10.1046/j.1523-1755.2003.00840.x, PMID: 12631062

[40] Gong DJ Wang L Yang YY Zhang JJ Liu XH. Diabetes aggravates renal ischemia and reperfusion injury in rats by exacerbating oxidative stress, inflammation, and apoptosis. Ren Fail. (2019) 41:750–61. doi: 10.1080/0886022X.2019.1643737, PMID: 31441362 PMC6720228

[41] Wang J Yue X Meng C Wang Z Jin X Cui X. Acute hyperglycemia may induce renal tubular injury through mitophagy inhibition. Front Endocrinol (Lausanne). (2020) 11:536213. doi: 10.3389/fendo.2020.536213, PMID: 33424763 PMC7793649

[42] Nie X Leng X Miao Z Fisher M Liu L. Clinically ineffective reperfusion after endovascular therapy in acute ischemic stroke. Stroke. (2023) 54:873–81. doi: 10.1161/STROKEAHA.122.038466, PMID: 36475464

[43] van den Akker JP Egal M Groeneveld AB. Invasive mechanical ventilation as a risk factor for acute kidney injury in the critically ill: a systematic review and meta-analysis. Crit Care. (2013) 17:R98. doi: 10.1186/cc12743, PMID: 23710662 PMC3706893

[44] Vemuri SV Rolfsen ML Sykes AV Takiar PG Leonard AJ Malhotra A. Association between acute kidney injury during invasive mechanical ventilation and ICU outcomes and respiratory system mechanics. Crit Care Explor. (2022) 4:e0720. doi: 10.1097/CCE.0000000000000720, PMID: 35782295 PMC9246080

[45] Frydman S Freund O Zornitzki L Banai S Shacham Y. Relation of mechanical ventilation to acute kidney injury in myocardial infarction patients. Cardiorenal Med. (2023) 13:263–70. doi: 10.1159/000533800, PMID: 37640019 PMC10664320

[46] Huang S Teng Y Du J Zhou X Duan F Feng C. Internal and external validation of machine learning-assisted prediction models for mechanical ventilation-associated severe acute kidney injury. Aust Crit Care. (2023) 36:604–12. doi: 10.1016/j.aucc.2022.06.001, PMID: 35842332

[47] Du R Wang L Wang Y Zhao Z Zhang D Zuo S. AKI prediction model in acute aortic dissection surgery: nomogram development and validation. Front Med (Lausanne). (2025) 12:1562956. doi: 10.3389/fmed.2025.1562956, PMID: 40443509 PMC12119464

