# Supplementary material for: A nomogram for predicting individual risk of acute kidney injury after endovascular therapy in large vessel occlusion stroke
Source: Front Med (Lausanne). 2025 Oct 8;12:1608293. doi: 10.3389/fmed.2025.1608293 (PMC12540481; doi:10.3389/fmed.2025.1608293)
Supplement: Supplementary file 1 [file Data_Sheet_1.docx]

**Supplementary Material 1:Comparison of ROC values before and after data imputation in sensitivity analysis**

**
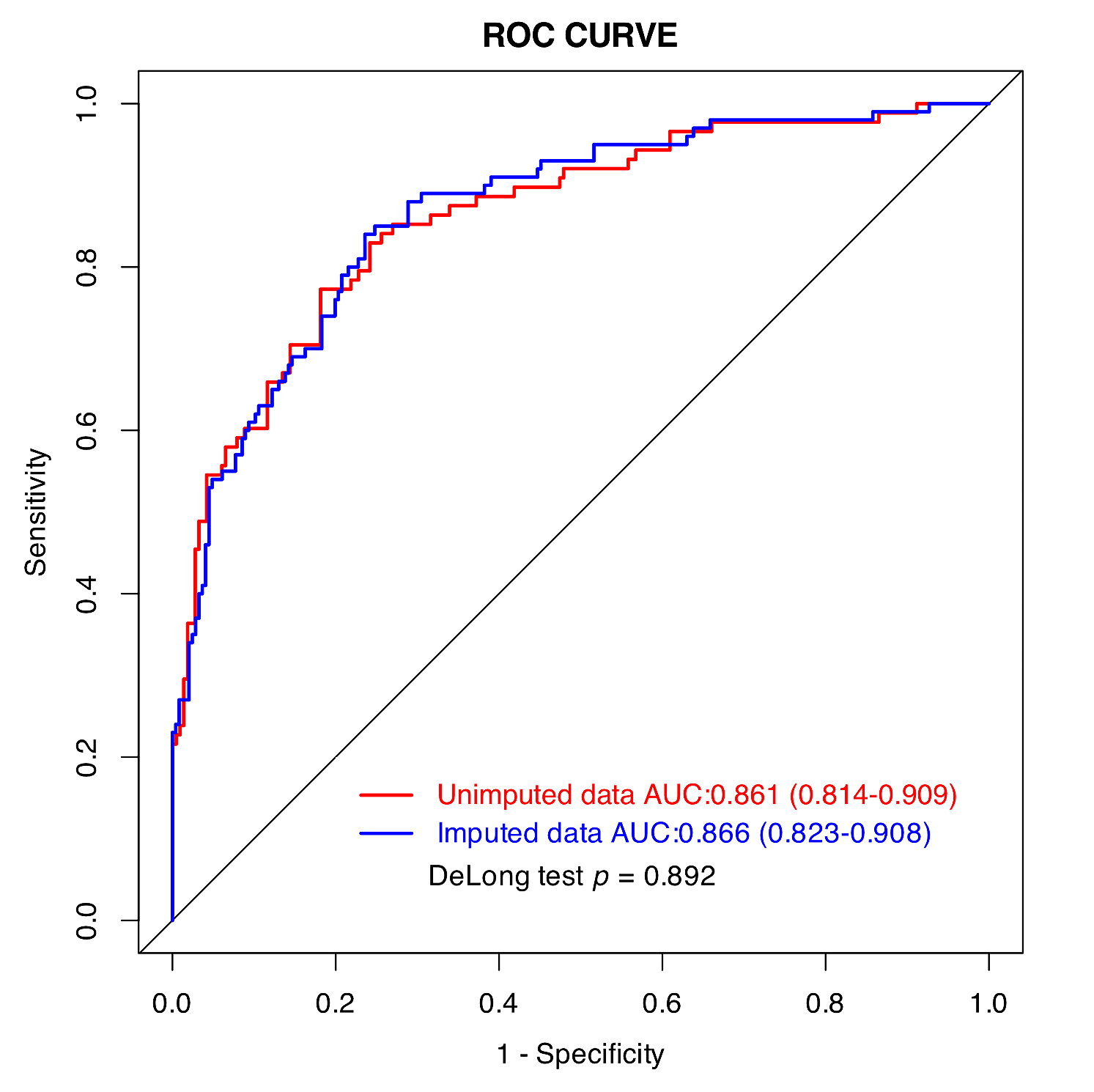
**

**Supplementary Material 2: Results of multicollinearity test**

| Variables | VIF | 1/VIF |
| --- | --- | --- |
| PLR | 7.831 | 0.128 |
| SII | 7.654 | 0.131 |
| SIRI | 1.758 | 0.569 |
| NLR | 5.527 | 0.181 |
| Operation_time | 1.099 | 0.910 |
| Days in ICU | 3.352 | 0.298 |
| Duration of MV | 3.209 | 0.312 |
| Admission blood glucose | 1.561 | 0.641 |
| Admission NIHSS | 3.830 | 0.261 |
| Admission GCS | 2.616 | 0.382 |
| Admission mRs | 2.080 | 0.481 |
| Systolic pressure | 2.027 | 0.493 |
| Diastolic pressure | 1.895 | 0.528 |
| Age | 1.761 | 0.568 |
| gender | 1.614 | 0.619 |
| Diabetes | 1.647 | 0.607 |
| Hypertension | 1.271 | 0.787 |
| Atrial fibrillation | 1.337 | 0.748 |
| Prior stroke | 1.184 | 0.844 |
| Heart failure | 1.163 | 0.860 |
| Smoking | 1.563 | 0.640 |
| Alcohol drinking | 1.449 | 0.690 |
| Cys C | 1.759 | 0.568 |
| WBC | 2.935 | 0.341 |
| Scr | 1.664 | 0.601 |
| proteinuria | 1.185 | 0.844 |
| ALB | 1.511 | 0.662 |
| UA | 1.312 | 0.762 |
| CRP | 1.267 | 0.789 |
| Postoperative complications | 1.218 | 0.821 |
| Mean VIF | 2.343 | . |
